# Perceived Stress and Associated Factors in Russian Medical and Dental Students: A Cross-Sectional Study in North-West Russia

**DOI:** 10.3390/ijerph17155390

**Published:** 2020-07-27

**Authors:** Sergei N. Drachev, Lina Stangvaltaite-Mouhat, Napat Limchaichana Bolstad, Jan-Are K. Johnsen, Tatiana N. Yushmanova, Tordis A. Trovik

**Affiliations:** 1 Department of Clinical Dentistry, Faculty of Health Sciences, UiT The Arctic University of Norway, N-9037 Tromsø, Norway; lina.stangvaltaite@uit.no (L.S.-M.); napat.l.bolstad@uit.no (N.L.B.); jan.a.johnsen@uit.no (J.-A.K.J.); 2 Department of Prosthodontics, Northern State Medical University, 163000 Arkhangelsk, Russia; yushmanowa.tatiana@yandex.ru; 3 Oral Health Centre of Expertise in Eastern Norway, 0369 Oslo, Norway; 4 Department of Community Medicine, Faculty of Health Sciences, UiT The Arctic University of Norway, N-9037 Tromsø, Norway; tordis.a.trovik@uit.no

**Keywords:** medical and dental students, perceived stress scale, minimal clinically important difference, Russia, Northern State Medical University

## Abstract

The aim was to assess perceived stress (PS) and factors associated with PS in Russian medical and dental students. A total of 406 medical and 283 dental students aged 18–25 years that attended the Northern State Medical University in Arkhangelsk, North-West Russia participated in this cross-sectional study. A structured, self-administered questionnaire was used to collect information on socio-demographic and socioeconomic factors, oral health (OH) behavior, and self-reported OH. All students were clinically examined to assess dental caries, oral hygiene, and gingiva. PS was measured by the Perceived Stress Scale 10 (PSS-10). Of the students, 26.0%, 69.1%, and 4.9% reported low, moderate, and high PS, respectively. Female sex (b = 2.28, 95% confidence interval (CI): 1.38–3.18), dental faculty (b = 1.74, 95% CI: 0.94–2.54), low subjective socioeconomic status (SES) (b = 1.71, 95% CI: 0.91–2.51), and irregular dental visits (b = 1.65, 95% CI: 0.72–2.58) were associated with higher PSS-10 score. These factors were assumed to be clinical meaningful, given that minimal clinically important difference of PSS-10 fell between 2.19 and 2.66 points. The majority of the medical and dental students reported moderate PS. Based on statistical significance and clinical meaningfulness, socio-demographic factors (sex, faculty), subjective SES, and OH behavior (regularity of dental visits) were associated with PS.

## 1. Introduction

In 1956, Hans Selye used the term “stress” to describe “the non-specific response of the body to any demand for change” [1]. Although stress is a normal biological reaction to a potentially dangerous mental, emotional, or physical exposure, chronic stress may result in depression, anxiety, burnout, and other negative health outcomes, including cardiovascular diseases, cold symptoms, and exacerbations of autoimmune diseases [2]. Oral health (OH) is an integral part of general health, and chronic stress may affect OH as well through individuals’ motivation to cope in unhealthy ways (poor diet, poor OH behavior, excessive use of alcohol and tobacco) [3]. Moreover, stress leading to the dysfunction of physiological systems [4] may play an important role in dental caries development [5]; progression of periodontal diseases and a poorer outcome of their treatment [4,6]. It has been found that high perceived stress is associated with poor self-reported OH, and the effect of stress is stronger for participants of lower socioeconomic position [3].

Several psychometric instruments have been designed to assess stress. The Perceived Stress Scale 10 (PSS-10) is a widely used tool to measure experienced stress in community samples with a junior high school education and higher [7,8]. This stress assessment is based on patient-reported outcome (PRO) measure, and although the main aim of a study is to detect differences between groups, testing statistical significance is insufficient to conclude whether these differences are clinically meaningful [9]. Therefore, the concept of minimal clinically important difference (MCID), which is defined as “the smallest change that is important to patients” [10], is particularly helpful to evaluate PROs [11]. There are two general approaches to determine the MCID: anchor-based and distribution-based methods [9,11,12]. The first one uses an external criterion based on other subjective or objective measures to compare PRO scores. The second approach compares the change in PRO scores to some measure of variability [9], such as the effect size (ES), the standard error of measurement (SEM), or one half of the standard deviation (SD) of baseline PRO scores [13]. Therefore, it is crucial to determine the MCID for PSS scores to interpret whether the observed group differences in perceived stress are clinically meaningful. Nevertheless, information on the clinical implications of differences in PSS scores is limited. We found only one study that analyzed PSS-10 scores in terms of MCID, estimated as 4.03 points and calculated as one half of SD, in patients with systemic lupus erythematosus [14]. 

Medical and dental students are particularly important groups in the study of perceived stress given a high level of stress during their training [15,16]. Academic pressure [17,18,19,20,21], clinical factors [18,20], psychosocial issues [17,19,21], financial problems [21], and environmental stressors [17] have been identified as potential main categories of stressors in medical and dental students. A higher stress was observed in females than in males [17,19,20], in dental students than in medical students [16,22]. A high level of daily stressors was found to be associated with poor self-reported OH and self-reported gum bleeding, dissatisfaction with teeth, and experienced tooth pain in dental students [23]. Academic stress has also an adverse effect on oral hygiene and clinically assessed gingival status in medical and dental students [24,25].

To our knowledge, there is little information on stress in Russian medical and dental students. One study investigated sources of stress in dental students in Arkhangelsk, North-West Russia [26]. Academic performance and high workload were found to be the main categories of stressors. Socio-demographic factors (female sex), socioeconomic factors (handle additional paid work during training), and clinical dentistry training are the significant predictors of the main sources of stress [26]. The study included only dental students and used a modified version of the Dental Environmental Stress questionnaire, which was originally developed to measure stress among dental students. In Russian medical and dental students, poor OH with high prevalence of dental caries and high DMFT index (i.e., the number of decayed teeth (DT), missing teeth (MT) due to dental caries, and filled teeth (FT)) was observed [27]. Moreover, 34% of the students skipped tooth-brushing sometimes during a week, every day or almost every day [27]. Nevertheless, no associations between stress and OH (both self-reported and clinically assessed) and OH behavior were studied among Russian medical and dental students. In addition, the conclusions of the aforementioned study [26] were drawn based on statistical significance, and no MCIDs in stress scores were calculated. 

**Figure 1:**
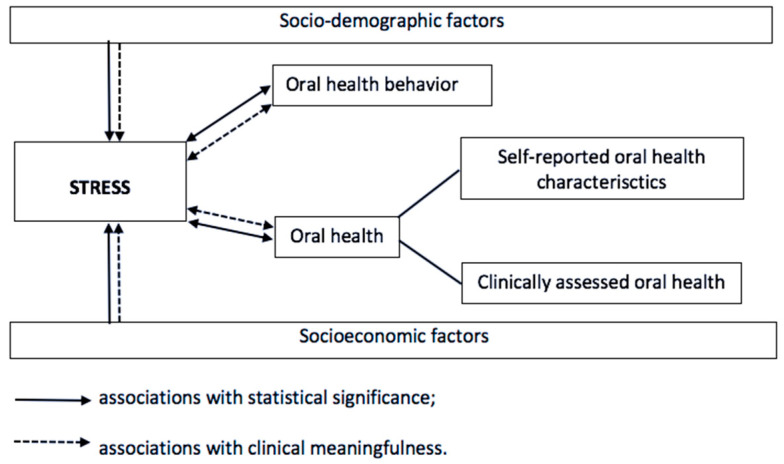
Conceptual framework of the study.

The present study aimed (1) to assess perceived stress measured by PSS-10 among Russian medical and dental students; (2) to investigate how socio-demographic and socioeconomic factors, OH behavior, and OH are related to perceived stress in the study sample from both statistical and clinical point of view. We hypothesized that high perceived stress among Russian medical and dental students is significantly associated with their poor OH (both self-reported and clinically assessed) and OH behavior, and these associations are clinically meaningful. Figure 1 reflects the conceptual framework of the study.

## 2. Materials and Methods

### 2.1. Study Design, Setting and Participants 

This cross-sectional study was carried out at the Northern State Medical University (NSMU), located in the city of Arkhangelsk, North-West Russia in the 2015–2016 academic year. We invited full-time undergraduate students from medical faculty, *n* = 1482, and from dental faculty, *n* = 524. For more details, see Drachev et al., 2017 [27].

### 2.2. Sampling

A two-stage sampling technique was applied for selection of medical and dental students. At Stage 1, all students from both faculties were informed verbally and in an invitation letter about the study and invited to participate at the end of a randomly selected curriculum lecture. Altogether, 78.7% of enrolled students attended the recruitment lectures, of whom 87.7% agreed to participate and signed the informed consent form. All students who agreed to participate in the study were asked to complete a structured, self-administered questionnaire 1 (Q1) and give their mobile phone numbers so we could contact them and set a date and time for Stage 2.

To achieve a comparable and representative sample, all dental students (*n* = 420) and a stratified, random, proportionate sample of medical students (*n* = 823) who participated in Stage 1 were invited to participate in Stage 2. All students participating in Stage 2 (474 medical and 333 dental students) completed a second, structured, self-administered questionnaire (Q2) and underwent a clinical dental examination. Non-Russian nationality, age under 18 or over 25 years, presence of fixed orthodontics bands, and pregnancy were considered the exclusion criteria for Stage 2. More details related to sampling, including sample size calculation for Stage 2, have been described elsewhere [27]. The students with no missing data in Q1 and Q2 (406 medical and 283 dental students) were included in analysis.

### 2.3. Questionnaires 

Q1 collected information on socio-demographic variables (age, sex, faculty, and place of childhood residence), OH behavior (regularity of dental visits, frequency of tooth-brushing, skipping tooth-brushing, and using toothpaste with fluoride), and self-reported OH characteristics (self-assessed OH, self-assessed dental aesthetic, satisfaction with mouth and teeth, experienced pain in mouth, experienced gum bleeding during tooth-brushing, and need for dental treatment).

Information on subjective socioeconomic status (SES), mother’s education, and perceived stress was obtained from Q2. To assess subjective SES, we applied the 10-step MacArthur Scale of Subjective Social Status [28]. The students were asked to report the ranking of their family in Russian society on a ladder with 10 rungs according to education, income, and occupation: 1 was “worst off” and 10 was “best off”. The list of variables with their categories used in Q1 and Q2 and in statistical analysis is presented in the Supplementary Materials (Table S1).

#### Perceived Stress as Measured by the Perceived Stress Scale-10: Validity and Reliability

The original English version of the PSS contains 14 items (PSS-14) on feelings and thoughts of respondents to evaluate the degree to which they appraise situations in their life as stressful [7]. Later, two shortened versions of the PSS with 10 items (PSS-10) and 4 items (PSS-4), which contain the items selected from the PSS-14, were developed [8]. The validity and reliability of PSS have been shown in many studies, and the psychometric properties of the PSS-10 were found to be superior compared with those of the PSS-4 and PSS-14 [29]. The instrument has been translated into more than 20 different languages, including Russian [30]. In fact, there are two Russian PSS versions which have slightly different wordings. One PSS-14 version is available online from the official website, but has not been previously tested in Russia [30]. Another PSS-10 version was translated from French and validated in Russian people aged 16–59 years [31]. The scale was shown to be a reliable and valid instrument for perceived stress assessment among Russian population [31]. Colleagues at the NSMU concluded that the two Russian versions have conceptual and functional equivalence. In the present study, the PSS-10 with the items taken from the official website was used [30].

The students were asked to indicate how often during the last month they felt or thought a certain way when faced with different situations in their life. Responses were given on a 5-point Likert scale: (“never” = 0, “almost never” = 1, “sometimes” = 2, “fairly often” = 3, and “very often” = 4). The total perceived stress score was calculated as the sum of all items in the PSS-10, with a range from 0 to 40 points. Items 4, 5, 7, 8 used reverse scoring and therefore they were recoded (0 = 4, 1 = 3, 2 = 2, 3 = 1, 4 = 0) before the total score calculation. A higher total score indicated a higher perceived stress. Although PSS is not a diagnostic instrument and there is no predetermined cut-off for PSS score [30], some studies used PSS scores of 0–13, 14–26, and 27–40 points to assess low, moderate, and high perceived stress, respectively [32,33,34].

That fact that only 25 of the 807 medical and dental students had missing values in the PSS-10 items adds support to the instrument’s face validity. PSS-10 scores distinguished significantly between study participants with good and poor self-assessed psychological health: overall mean PSS-10 score in the categories of excellent, very good, and good psychological health was 16.0, whereas the corresponding figure in the categories of fair and poor psychological health was 21.7, implying a good construct validity. After recording items 4, 5, 7, 8, Cronbach’s alpha for the total scale scores was 0.83, which reflects good internal consistency of the PSS-10 items. Moreover, removal of any item would result in a lower Cronbach’s alpha. No negative correlations between the PSS-10 items were found, and the mean inter-item correlation was 0.32 (range: 0.10–0.55). The corrected item-total correlations ranged from 0.39 to 0.65; these values were greater than 0.20, which demonstrated that all PSS-10 items met criterion for inclusion into a scale [35].

### 2.4. Assessment of Minimal Clinically Important Difference in PSS-10 Score

Both anchor-based and distribution-based approaches were used to assess MCID of PSS-10 score. For the anchor-based approach, in Q2, students were asked the one global item about their psychological health, which was applied as the external reference criterion. Response options included “excellent”, “very good”, “good”, “fair”, and “poor”. Differences in mean PSS-10 scores between the adjacent responses of the anchor were calculated. The mean of the mean differences provided the MCID estimate [9]. Spearman rank correlation between the anchor responses and the PSS-10 score was greater than 0.3 (0.44, *p* < 0.001) that allowed to include the anchor in MCID calculations [12].

For the distribution-based approach, we used the two following methods: (1) one-half standard deviation (SD) [11,13]; (2) the standard error of measurement (SEM) [11]. The first method, which was suggested by Norman et al. [13], implies that 0.5 SD corresponds to MCID and is equal to an effect size (ES) of 0.5 [11]. The ES is a standardized measure, and values of 0.2, 0.5, and 0.8 are conventionally interpreted as small, moderate, and large effect, respectively [36]. For the second method, the SEM, which is defined as the variability between an individual’s observed and true score, was calculated as the product of the SD of the measure and the square root of 1 – its reliability [12]. For the SEM calculation, we used Cronbach’s alpha as the measure of reliability of the PSS-10 scores. One SEM is supposed to be MCID, and a difference smaller than one SEM is considered a measurement error [11].

### 2.5. Clinical Examination

A noninvasive clinical dental examination without radiographs was carried out by one dentist (S.N.D.), who was calibrated on examination technics according to World Health Organization (WHO) recommendations [37] at the Dental Clinic of UiT The Arctic University of Norway, Tromsø, Norway, before the study start. We calculated the DMFT index taking into account all permanent teeth, excluding third molars. For analysis, we dichotomized DT and MT as non DT/one or more DT and non MT/one or more MT, respectively. Given the skewed distribution of FT and DMFT index and using the median FT and the median DMFT index (7.0 for both variables) as the cut-offs, FT and DMFT index were categorized as low (less than 7) and high (7 and more). To assess oral hygiene, the Simplified Oral Hygiene Index (OHI-S) of Green and Vermillion (1964) was used [38]. The OHI-S from 0 to 1.2, from 1.3 to 3.0, and from 3.1 to 6.0 was considered good, fair, and poor oral hygiene, respectively [38]. Given the maximum value of 3.0 in our study sample, we used the two categories of the OHI-S (good and fair oral hygiene) for analysis. For gingiva assessment, we applied the Gingival Index (GI) proposed by Loe and Silness (1963) [39]. Four areas (buccal, lingual, mesial, and distal) of each of the six index teeth (44/32/36/24/12/16) were considered for GI calculation. All clinical dental examinations were carried out at the Dental Clinic of the NSMU from February to May 2016. In June 2016, 54 randomly selected students who participated in Stage 2 underwent clinical dental re-examination. For DMFT index and GI, intraclass correlation coefficients were 0.989 (95% confidence interval (CI): 0.981–0.993) and 0.828 (95% CI: 0.721–0.896), respectively. For DT /non DT teeth, the calculated value of Kappa statistic was 0.804 (95% CI: 0.641–0.967), implying a strong agreement [40].

### 2.6. Statistical Analysis

For missing values analysis, Little’s missing completely at random (MCAR) test was used [41]. Simple linear regression models were constructed to find crude associations between dependent variable (PSS-10 score) and independent variables (socio-demographic and socioeconomic factors, OH behavior, and OH). Predictors with *p*-values < 0.2 in univariable analysis were used in the multivariable linear regression model. Backward stepwise selection was applied to find adjusted associations between PSS-10 score and independent variables. Predictors with *p*-values ≥ 0.2 and *p*-values < 0.1 were eligible for removal and addition to the final model, respectively. The final linear regression model was checked for normality of residuals, homoscedasticity, outlier impact, and multicollinearity, and no violations of these assumptions were found. Unstandardized b coefficients with 95% CI, t-statistics, and *p*-values were used to present the results of multivariable analysis. All statistical tests were two-sided, and the significance level was set at 5%.

IBM SPSS Statistics version 26.0 (IBM Corp., Armonk, NY, USA) and STATA version 15.0 (StataCorp, College Station, TX, USA) were used for statistical analysis.

### 2.7. Ethics

The Regional Ethical Committee of Norway and the Ethical Committee of the NSMU, Russia approved the study protocol (2015/1788/REK nord and № 05/10-15 from 19.10.2015, respectively).

## 3. Results

Of the 807 students who answered the PSS-10, one did not answer all items, 20 omitted one item, and 4 students omitted two items. No significant differences across all independent variables between students with missing PSS-10 data (*n* = 25) and those without missing PSS-10 data (*n* = 782) were found. Moreover, Little’s MCAR test was insignificant (*p* = 0.301) for the set of variables with missing values included in analysis. Assuming that the data are MCAR, we applied a complete-case analysis, where only students with no missing data in Q1 and Q2 were considered in the statistical analysis (406 medical and 283 dental students).

**Figure 2:**
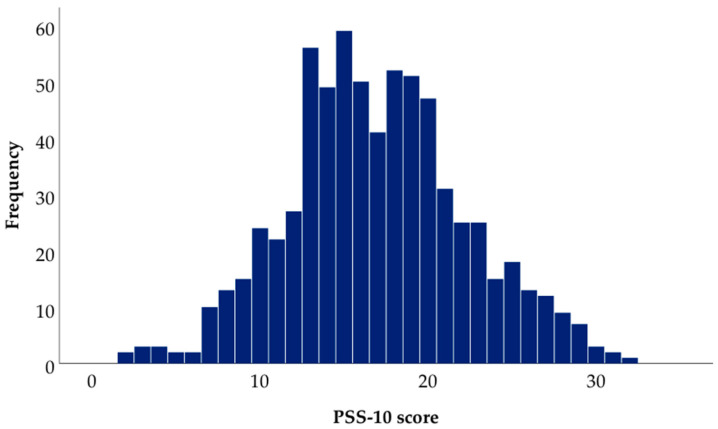
Histogram of the Perceived Stress Scale 10 (PSS-10) score in the overall study sample (*n* = 689).

In the study sample, the mean PSS-10 score was 16.97 (SD 5.31; 95%CI: 16.57–17.36; range: 30, from 2 to 32) (Figure 2). Overall, 26.0%, 69.1%, and 4.9% of the students reported low, moderate, and high perceived stress, respectively. Mean age of the students was 20.2 years (SD 1.6). Seventy-five percent of the students were women, 72.0% reported an urban place of childhood residence, and 54.3% had a mother with a university education. The mean PSS-10 score was higher in women than in men; in those who reported low subjective SES (less than 6) than in those who reported high subjective SES (6 and more). There were no differences in mean PSS-10 score in different age groups or between categories of place of childhood residence and mother’s education. Dental students reported a higher perceived stress compared with medical students (17.44 vs. 16.64), although the differences did not reach statistical significance (Table 1).

When looking at OH behavior, the majority of the students reported regular dental visits and frequent tooth-brushing (78.1% and 80.3%, respectively). Nevertheless, one-third of the study participants reported skipping tooth-brushing. Nearly half of the students used a toothpaste with fluoride. Students who visited a dentist irregularly had a higher PSS-10 score compared with those who visited a dentist regularly. No differences in the PSS-10 score were observed between categories of other OH behavior variables (Table 2).

Overall, 63.1% and 61.1% of the students had good self-assessed OH and self-assessed dental aesthetic. More than half of the students were dissatisfied with their mouth and teeth or had difficulties to answer. Nearly half of the study participants experienced pain in their mouth and gum bleeding during tooth-brushing. Moreover, 58.2% of the students reported need for dental treatment and 11.9% of the students had difficulties to answer. Students with poor self-reported OH characteristics had a higher PSS-10 score (Table 3).

In the study sample, the mean DMFT index was 7.65 (SD 4.47; range 27, from 0 to 27). FT were the main component of the DMFT index (90.6%). The number of DT, MT, and FT varied from 0 to 9, from 0 to 3, and from 0 to 22, respectively. Altogether, 32.5% and 9.4% of the students had one and more DT and one and more MT, respectively. The majority of students (63.7%) had good oral hygiene. The mean GI was 0.28 (SD 0.24; range 1.29, from 0 to 1.29). There were no significant differences in the PSS-10 score between categories of clinically assessed OH (Table 4).

Multivariable linear regression with PSS-10 score as the dependent variable showed that female sex, dental faculty, low subjective SES, irregular dental visits, using toothpaste without fluoride/difficult to answer, experienced pain in mouth, and poor self-assessed dental aesthetic were associated with higher PSS-10 score. For example, the adjusted mean PSS-10 score in students who reported irregular dental visits was higher by 1.65 (95% CI: 0.72–2.58) points than that found in those who reported regular dental visits. Based on t-statistics and *p*-values, the most important predictors of higher perceived stress were socio-demographic (sex, faculty), socioeconomic (subjective SES) factors, and OH behavior (regularity of dental visits) (Table 5).

According to the anchor-based approach, the MCID, calculated as the mean of the mean differences between the adjacent responses of the anchor, was 2.43 points (the mean differences between the adjacent responses varied from 1.23 to 4.12 points). When applying the distribution-based approach, the MCIDs, calculated as the 0.5 SD and SEM, were 2.66 points and 2.19 points, respectively. Based on these MCID calculations and the adjusted unstandardized b coefficients with their 95% CIs, one may assume that socio-demographic factors (female sex, dental faculty), socioeconomic factors (low subjective SES), and OH behavior (irregular dental visits) had not only statistical significance, but also clinical importance in relation to the outcome studied. Other statistically significant factors (using toothpaste without fluoride/difficult to answer, experienced pain in mouth, poor self-assessed dental aesthetic) were assumed to be clinically nonsignificant factors associated with higher perceived stress in medical and dental students.

## 4. Discussion

In the current study, we found that 69.1% of Russian medical and dental undergraduate students that attended the NSMU in Arkhangelsk, North-West Russia reported moderate perceived stress measured by PSS-10. The mean PSS-10 score was 16.6 and 17.4 in medical and dental students, respectively. Socio-demographic factors (female sex, dental faculty), socioeconomic factors (low subjective SES), OH behavior (irregular dental visits, using toothpaste without fluoride/difficult to answer), and self-reported OH characteristics (experienced pain in mouth, poor self-assessed dental aesthetic) were statistically significant factors associated with higher PSS-10 score. Our analysis suggested the MCID of PSS-10 likely falls between the values of 2.19–2.66 points. Sex, faculty, subjective SES, and regularity of dental visits may have clinical meaningfulness in relation to perceived stress.

We did not find other studies among medical and dental students that presented a prevalence of low, moderate, and high perceived stress using the same cut-offs for PSS-10 score that were applied in our study. Nevertheless, the mean PSS-10 score among medical students in the present study (16.6) was lower than that reported in medical students in Malaysia (19.5) [42], Singapore (19.0 in 2016 and 17.6 in 2017) [43], and Korea (18.6) [44], and was higher than that found in Thailand (13.5) [45]. In dental students, corresponding figures in our study (17.4) were found to be lower than that reported in Romania (20.6) and Malaysia (21.7), and comparable to that observed in England, South Africa, Australia, United States, and Greece [46]. One obvious explanation of this finding is that medical and dental training environments and curricula differ across countries. Moreover, social, economic, and cultural factors may play an important role in stress perception. A study among college students aged 20–25 years found that Russian students showed a higher level of perceived stress and a less frequent incidence of reported somatic illness than their American counterparts [47]. When explaining these differences, researchers assumed that Russians may be more pessimistic, lethargic, and defensive or may experience more stressors in everyday life than Americans [47]. This study was conducted in 2000, and the socioeconomic situation in Russia has been changed positively from that time. Nevertheless, 58.2% of our medical and dental students reported need for dental treatment, whereas only 32.5% of the students had untreated carious lesions, and these findings, to some extent, may reflect a pessimistic orientation of the study participants. To better understand stress perception by medical and dental students in different cultural settings, further multi-country studies should be designed and conducted. 

As we hypothesized and in agreement with prior studies [23], our study has shown that poor OH behavior and poor self-reported OH characteristics are significantly associated with higher perceived stress. The model, postulated by Vasiliou et al. in 2016, on causal and moderating pathways linking stress to OH practices and outcomes hypothesized that behaviors to cope with chronic stress may manifest as unhealthy habits with respect to OH, leading to oral diseases [3]. Although causality cannot be established in the present study, one may speculate that our findings are consistent with this model. Indeed, students with high perceived stress may delay dental visit, which in turn may lead to more extensive OH problems, including pain in mouth and poor dental aesthetic. Our analysis has also shown that from all variables related to OH behavior and self-reported OH characteristics, irregular dental visits is the most important predictor of higher perceived stress, and the association may have clinical meaningfulness. Nevertheless, we did not find differences in the PSS-10 score between categories of clinically assessed OH. One possible explanation for this finding is that students appraised situations in their life as stressful during the last month, whereas dental caries is a slow disease, and more than one month is needed for its development. In addition, dental caries experience, expressed using the DMFT index, is an accumulative indicator of poor OH. In our study sample, FT were the main component of the DMFT index (90.6%), and this fact is in agreement with the finding that the majority of the students (78.1%) visited a dentist regularly. Although the students with high FT had a lower stress compared with those with low FT, these differences were not statistically significant. Nonetheless, in contrast, an Indian study demonstrated a weak statistically significant correlation between PSS score and DMFT index in students aged 15–18 years [48]. Studies also found significant associations between stress, plaque, and gingivitis in medical and dental students who had university exams [24,25]. In our study, all clinical dental examinations were performed from February to May, when the students did not have any exams, which, to some extent, might explain our findings. 

We found that the dental students had a higher perceived stress than the medical students, which is consistent with findings from other studies [16,22,49]. One may speculate that five-year dental education which combines obtaining theoretical knowledge and learning practical skills including the ability to use specific dental equipment; high concentration of dental students when they perform complex irreversible dental procedures, often independently; and communication with fearful dental patients may result in a higher stress level in dental students than in medical students. Indeed, a comparative study of professional student stress showed that dental students reported higher levels of perceived stress than medical students in the categories of academic performance, patient and clinic responsibilities, and faculty relations [22].

Female students showed a higher PSS-score than male students, which is in line with results from other studies [17,19,20,43,46,50]. Females may express their experience of stress more openly than males [46,51]. Moreover, compared with male students, female students may be more sensitive to certain environmental stressors or may have a different pattern of educational morbidity due to different reaction to stress [51]. Researchers explain this fact through a complex biological nature of the sex-specific stress response, which may be dependent on hormonal fluctuations; sex differences in brain structure, white matter organization, and cerebral blood flow; and engagement of distinct neural networks in men and women during stress [52,53].

In the present study, a lower subjective SES was associated with higher PSS-scores, which is in line with other reports [54,55]. In 2007, Taiwanese researchers found that medical students whose mothers had a lower SES reported a higher stress [54]. In 2012, a Colombian study also observed a significant association between a lower subjective SES and a higher level of psychological distress in a large cohort of dental students [55]. One may speculate that distal extracurricular factors, such as SES, are important and independent determinants of perceived stress in medical and dental students. Individuals with a lower SES may have lack of flexibility to cope with stressful situations. Other possible impacts of low SES include “lower self-esteem, blocked aspirations, status frustrations, impaired efficacy, fatalism and lower mastery and personal control” [54], which in turn may lead to a higher level of perceived stress. Interestingly, a similar association between SES and stress may also be assumed in Russian medical doctors and dentists. A survey showed that 53.1% and 30.0% of Russian medical doctors assessed their social status as average and below average, respectively. Low income and excessive fatigue were found to be the most important factors, which have a negative impact on the study participants and their family [56]. Another study conducted among 175 Russian dentists showed that 76.3% of dentists reported signs of burnout, whereas only 5.7% of the respondents perceived themselves to belong to a group of high social status [57]. National policy should focus on increasing SES of Russian healthcare professionals, including medical and dental students, to reduce perceived stress and improve their psychological well-being.

This study conducted in North-West Russia was the first attempt to assess perceived stress measured by PSS-10 and associated factors, including OH and OH behavior, in Russian medical and dental students. The results of the present study provide evidence of good internal consistency of the PSS-10 items, good face and construct validity of the instrument. To assess difference in perceived stress that the students would consider important, MCID of the PSS-10 was calculated using both anchor-based and distribution-based approaches. All study participants underwent clinical dental examination, and good consistency of the obtained clinical data was shown. Nevertheless, the results of the study should be interpreted with caution, taking into consideration the following limitations. That fact that only medical and dental students from one medical university located in North-West Russia were included in the study may limit the extrapolation of our findings to all Russian medical and dental students. Given the cross-sectional design of the present study, we cannot establish causality between the studied variables and demonstrate a trend in the prevalence of high stress over time. Moreover, when assessing MCID, our study identified between-group differences in PSS-10 score and did not evaluate within-subjects changes over time. Test–retest reliability of PSS-10 was not assessed. For the anchor-based approach, we applied one global item asking students how they rate their psychological health, which may not reflect an importance of differences in perceived stress. Moreover, a single item is assumed to be a less valid and reliable measure than a multi-item instrument [58]. In addition, the choice of anchor’s categories may have a marked effect on MCID values [9]. In our study, the mean differences between the adjacent responses varied from 1.23 to 4.12 points, and to estimate MCID we calculated the mean of these differences. Although this approach was previously described as a potential solution when choosing anchor’s categories, much more empirical evidence is needed for the applicability of this method [9]. Finally, to confirm findings from the study, it is strongly recommended to apply multiple independent anchors across multiple samples [12]. For the distribution-based approach, there is no theoretical explanation why one-half SD or one SEM are MCID [58]. It is also suggested that distribution-based methods assess minimal detectable change, rather than MCID [58]. Moreover, to assess SEM we used the Cronbach’s alpha calculated for all PSS-10 items, assuming unidimensionality of PSS-10 [8]. Nevertheless, some studies showed a multidimensional nature of PSS-10 that may complicate interpretation of the results based on all 10 items [59]. During the clinical dental examination, we used only visual and tactile methods and did not take radiographs; thus, an underestimation of dental caries cannot be excluded. In the present study, information on OH behaviors, SES, dental aesthetic, and psychological health was self-reported; therefore, under- or over-reporting bias cannot be ignored.

## 5. Conclusions

The majority of the medical and dental undergraduate students that attended the NSMU in Arkhangelsk, North-West Russia reported moderate perceived stress. Socio-demographic factors (female sex, dental faculty), socioeconomic factors (low subjective SES), OH behavior (irregular dental visits, using toothpaste without fluoride/difficult to answer), and self-reported OH characteristics (experienced pain in mouth, poor self-assessed dental aesthetic) were statistically significant factors associated with higher perceived stress. In terms of MCIDs, sex, faculty, subjective SES, and regularity of dental visits were assumed to be clinically meaningful. No significant associations between stress and clinically assessed OH were found. Medical and dental educators need to be aware on those groups of students who have a higher perceived stress. Administrative measures should focus on developing preventive strategies for stress management to improve students’ psychological well-being. In addition, coping methods used by Russian medical and dental students should also be investigated. 

## Figures and Tables

**Table 1:** Socio-demographic and socioeconomic factors associated with perceived stress in the study sample.

Variable	*n* (%)	Crude Unstandardized b Coefficient (95% CI)	*p* ^1^
**Age group (years)**			0.183
18–20	404 (58.6)	Reference	
21–25	285 (41.4)	−0.55 (−1.35; 0.26)	
**Sex**			<0.001
Male	170 (24.7)	Reference	
Female	519 (75.3)	1.89 (0.98; 2.80)	
**Faculty**			0.052
Medical	406 (58.9)	Reference	
Dental	283 (41.1)	0.80 (−0.01; 1.61)	
**Place of childhood residence**			0.967
Urban	496 (72.0)	Reference	
Rural	193 (28.0)	−0.02 (−0.90; 0.87)	
**Subjective SES**			<0.001
High (6 and more)	460 (66.8)	Reference	
Low (less than 6)	229 (33.2)	1.82 (0.99; 2.66)	
**Mother’s education**			0.801
University	374 (54.3)	Reference	
Lower than university	315 (45.7)	0.10 (−0.70; 0.90)	

Abbreviations: SES, socioeconomic status; CI, confidence interval; ^1^ *p*-values from simple linear regression models (Perceived Stress Scale 10 score was the dependent variable).

**Table 2:** Oral health behavior associated with perceived stress in the study sample.

Variable	*n* (%)	Crude Unstandardized b Coefficient (95% CI)	*p* ^1^
**Regularity of dental visits**			<0.001
Regular	538 (78.1)	Reference	
Irregular	151 (21.9)	2.01 (1.06; 2.96)	
**Tooth-brushing**			0.739
Frequent	553 (80.3)	Reference	
Infrequent	136 (19.7)	0.17 (−0.83; 1.17)	
**Skipping tooth-brushing**			0.073
No	459 (66.6)	Reference	
Yes	230 (33.4)	0.77 (−0.07; 1.61)	
**Toothpaste**			0.127
With fluoride	321 (46.6)	Reference	
Without fluoride/difficult to answer	368 (53.4)	0.62 (−0.18; 1.42)	

Abbreviations: CI, confidence interval; ^1^ *p*-values from simple linear regression models (Perceived Stress Scale 10 score was the dependent variable).

**Table 3:** Self-reported oral health characteristics associated with perceived stress in the study sample.

Variable	*n* (%)	Crude Unstandardized b Coefficient (95% CI)	*p* ^1^
**Self-assessed oral health**			0.014
Good	435 (63.1)	Reference	
Poor	254 (36.9)	1.03 (0.21; 1.85)	
**Self-assessed dental aesthetic**			<0.001
Good	421 (61.1)	Reference	
Poor	268 (38.9)	1.73 (0.92; 2.53)	
**Satisfaction with mouth and teeth**			<0.001
Yes	302 (43.8)	Reference	
No/difficult to answer	387 (56.2)	1.66 (0.86; 2.45)	
**Experienced pain in mouth**			<0.001
No	374 (54.3)	Reference	
Yes	315 (45.7)	1.63 (0.84; 2.42)	
**Experienced gum bleeding during tooth-brushing**			0.017
No	392 (56.9)	Reference	
Yes	297 (43.1)	0.97 (0.17; 1.77)	
**Need for dental treatment**			0.030
No	206 (29.9)	Reference	
Yes/difficult to answer	483 (70.1)	0.96 (0.09; 1.82)	

Abbreviations: CI, confidence interval; ^1^ *p*-values from simple linear regression models (Perceived Stress Scale 10 score was the dependent variable).

**Table 4:** Clinically assessed oral health associated with perceived stress in the study sample.

Variable	*n* (%)	Crude Unstandardized b Coefficient (95% CI)	*p* ^1^
**Decayed teeth**			0.720
Non decayed teeth	465 (67.5)	Reference	
One and more decayed teeth	224 (32.5)	0.16 (−0.69; 1.00)	
**Missing teeth**			0.062
Non missing teeth	624 (90.6)	Reference	
One and more missing teeth	65 (9.4)	1.29 (−0.06; 2.65)	
**Filled teeth**			0.068
Low (less than 7)	329 (47.8)	Reference	
High (7 and more)	360 (52.2)	−0.74 (−1.53; 0.06)	
**DMFT index**			0.312
Low (less than 7)	294 (42.7)	Reference	
High (7 and more)	395 (57.3)	−0.41 (−1.22; 0.39)	
**OHI-S**			0.435
Good oral hygiene	439 (63.7)	Reference	
Fair oral hygiene	250 (36.3)	0.33 (−0.50; 1.16)	
**GI^2^**		−0.84 (−2.48; 0.80)	0.313

Abbreviations: DMFT, decayed, missing, and filled teeth; OHI-S, Simplified Oral Hygiene Index; GI, Gingival Index; ^1^ *p*-values from simple linear regression models (Perceived Stress Scale score 10 was the dependent variable); ^2^ GI was used as a continuous variable.

**Table 5:** Associations between perceived stress and selected variables in the study sample: results from multivariable linear regression ^1^.

Variable	t-Statistic	Adjusted Unstandardized b Coefficient (95% CI)	*p*
**Age group (years)**	−1.79		0.074
18–20		Reference	
21–25		−0.70 (−1.46; 0.07)	
**Sex**	4.97		<0.001
Male		Reference	
Female		2.28 (1.38; 3.18)	
**Faculty**	4.29		<0.001
Medical		Reference	
Dental		1.74 (0.94; 2.54)	
**Subjective SES**	4.19		<0.001
High (6 and more)		Reference	
Low (less than 6)		1.71 (0.91; 2.51)	
**Regularity of dental visits**	3.48		0.001
Regular		Reference	
Irregular		1.65 (0.72; 2.58)	
**Toothpaste**	2.42		0.016
With fluoride		Reference	
Without fluoride/difficult to answer		0.94 (0.18; 1.71)	
**Skipping tooth-brushing**	1.39		0.166
No		Reference	
Yes		0.59 (−0.25; 1.42)	
**Experienced pain in mouth**	2.74		0.006
No		Reference	
Yes		1.12 (0.32; 1.92)	
**Experienced gum bleeding during tooth-brushing**	1.31		0.189
No		Reference	
Yes		0.51 (−0.25; 1.28)	
**Self-assessed dental aesthetic**	2.26		0.024
Good		Reference	
Poor		1.00 (0.13; 1.86)	
**Satisfaction with mouth and teeth**	1.46		0.146
Yes		Reference	
No/difficult to answer		0.63 (−0.22; 1.48)	
**Missing teeth**	1.52		0.128
Non missing teeth		Reference	
One and more missing teeth		1.01 (−0.29; 2.31)	
**Filled teeth**	–1.92		0.056
Low (less than 7)		Reference	
High (7 and more)		–0.75 (−1.52; 0.02)	

Abbreviations: SES, socioeconomic status; CI, confidence interval. ^1^ the final regression model with backward stepwise selection of variables (Perceived Stress Scale score 10 was the dependent variable); R-squared = 15.0%.

## Supplementary Materials

The following is available online at https://www.mdpi.com/1660-4601/17/15/5390/s1, Table S1: The list of variables with their categories used in original questionnaires and in statistical analysis.
