# Supplementary material for: Perceived Stress and Associated Factors in Russian Medical and Dental Students: A Cross-Sectional Study in North-West Russia
Source: Int J Environ Res Public Health. 2020 Jul 27;17(15):5390. doi: 10.3390/ijerph17155390 (PMC7432408; doi:10.3390/ijerph17155390)
Supplement: Supplementary file 1 [file ijerph-17-05390-s001.pdf]

Table S1. The list of variables with their categories used in original questionnaires and in statistical analysis.

Group of variables	Variable	Categories in original questionnaires	Categories in statistical analysis
Socio-demographic	Age	Quantitative variable	18-20 years
			21-25 years
	Sex	Male	Male
		Female	Female
	Faculty	Medical	Medical
		Dental	Dental
	Place of childhood residence	Urban	Urban
		Rural	Rural
Socioeconomic	Subjective socioeconomic status	Quantitative variable (from 1 to 10)*	High (6 and more)
			Low (less than 6)
	Mother's education	University	University
		High school (9-11 years)	Low than university
		Specialized secondary	
		Difficult to answer**	-
Oral health behavior	Regularity of dental visits	Regularly, at least once every 6 months	Regular
		Regularly, at least once a year	
		Occasionally	Irregular
		No visits in the last 3 years	
		Difficult to answer**	-
	Frequency of tooth-brushing	Never	Infrequent
		Less than once a week	
		Once every few days	
		Once a day	
		Twice a day	Frequent
		More than twice a day	
	Skipping tooth-brushing	Never or almost never	No
		Sometimes during a week	Yes
		Every day or almost every day	
	Toothpaste	With fluoride	With fluoride
		Without fluoride	Without fluoride/ difficult to answer
		Difficult to answer	
Self-reported oral health characteristics	Self-assessed oral health	Excellent	Good
		Very good	
		Good	
		Fair	Poor
		Poor	
		Difficult to answer**	-
	Self-assessed dental aesthetic	Excellent	Good
		Very good	
		Good	
		Fair	Poor
		Poor	
		Difficult to answer**	-
	Satisfaction with mouth and teeth	Yes	Yes
		No	No/difficult to answer

		Difficult to answer	
	**Experienced pain in mouth**	Never	No
		Rarely	
		Sometimes	Yes
		Often	
		Always	
		Difficult to answer**	-
	**Experienced gum bleeding during tooth-brushing**	Never	No
		Rarely	
		Sometimes	Yes
		Often	
		Always	
		Difficult to answer**	-
	**Need for dental treatment**	No	No
		Yes	Yes/difficult to answer
		Difficult to answer	

* The variable "Subjective socioeconomic status" was split into two categories given the skewed distribution and the median value of 6.0;

** The students who chose the option "difficult to answer" (n=54 in questionnaire 1 and n=7 in questionnaire 2) were excluded from statistical analysis.
